# Prescribed safer supply during dual public health emergencies: a qualitative study examining service providers perspectives on early implementation

**DOI:** 10.1186/s13011-024-00598-7

**Published:** 2024-03-05

**Authors:** J. McCall, H. Hobbs, C. Ranger, F. Cameron, H. Stuart, J. Nelken, J. Majalahti, K. Urbanoski, G. Kolla, J. LeMaistre, K. Toombs, R. Herriot, Bernie Pauly

**Affiliations:** 1 University of Victoria, PO Box 1700, Stn CSC, Victoria, BC V8W 2Y2 Canada; 2 Canadian Institute of Substance Use Research, 2300 McKenzie Ave, Victoria, BC V8N 5M8 Canada; 3 SOLID Outreach, 1056 North Park St, Victoria, BC Canada; 4 AVI Health and Community Services, 713 Johnson Street, Victoria, BC V8W 1M8 Canada; 5 Victoria SAFER Program, 1806 Cook Street, Victoria, BC V8W 1M8 Canada

**Keywords:** Overdose, Prescribed safer supply, Safer supply, Qualitative research, Implementation science, Service providers, Toxic drug crisis, Harm reduction

## Abstract

**Background:**

Within North America and worldwide, drug related overdoses have increased dramatically over the past decade. COVID-19 escalated the need for a safer supply to replace unregulated substances and to reduce toxicity and overdoses. Service providers play an integral role in the delivery of safer supply but there is little empirical evidence that conceptualizes effective safer supply from their perspectives. This study explored early implementation and impacts of a safer supply program, capturing the perspectives of an interdisciplinary team of service providers on tensions and issues encountered in the development of the SAFER program.

**Methods:**

Using a community-based participatory approach, we conducted individual interviews with program providers (*n* = 9). The research team was composed of researchers from a local drug user organization, a local harm reduction organization, and academic researchers. The Consolidated Framework for Implementation Research (CFIR) informed the interview guide. Data was analyzed using thematic analysis.

**Results:**

There are six themes describing early implementation: (1) risk mitigation prescribing as context for early implementation; (2) developing SAFER specific clinical protocols; (3) accessibility challenges and program innovations; (4) interdisciplinary team and wraparound care; (5) program tensions between addiction medicine and harm reduction; (6) the successes of safer supply and future visions.

**Conclusion:**

Early implementation issues and tensions included prescriber concerns about safer supply prescribing in a highly politicized environment, accessibility challenges for service users such as stigma, encampment displacement, OAT requirements, program capacity and costs, and tensions between addiction medicine and harm reduction. Navigating these tensions included development of clinical protocols, innovations to reduce accessibility challenges such as outreach, wraparound care, program coverage of medication costs and prescribing safer supply with/without OAT. These findings contribute important insights for the development of prescribed safer supply programs.

## Background

Deaths due to drug toxicity (overdoses) have reached unprecedented levels in the past decade in North America and parts of Europe. In Canada, more than 36,000 people have died of apparent drug toxicity since 2016 [1]. In British Columbia (BC), Canada, a public health emergency was called in 2016 [2], yet the rate of drug toxicity deaths has continued to escalate and worsened with the onset of the COVID-19 pandemic. In 2021, there was a record 2,306 drug toxicity deaths, equating to 6.1 deaths per day in BC – a 26% increase from 2020 [3]. In 2022, 2,383 deaths due to unregulated drugs were reported in BC with rates continuing to rise to 2511 in 2023 [4]. This crisis has resulted from an increasingly toxic and volatile unregulated drug supply, contaminated with fentanyl and its analogues, combined with benzodiazepines and other non-opioid sedatives [3].

Prior to COVID-19, Health Canada funded a small number of prescriber-based safer supply programs [5, 6]. Prescribed safer supply programs use prescriptions to provide pharmaceutical alternatives to the unregulated and toxic drug supply. The main purpose of prescribed safer supply is the use of a harm reduction approach that aims to reduce overdoses and related harms, distinguishing safer supply conceptually from treatment interventions such as opioid agonist therapy (OAT). Prescribed safer supply may include medication types and formulations (e.g., oral, injectable) typically not available through OAT, and may be offered through program models with relatively more flexibility and fewer restrictions [7, 8]. In practice, however, the distinctions between OAT and prescribed safer supply are not always clear. For example, some authors refer to heroin assisted therapy, tablet and injectable opioid agonist therapy (TiOAT and iOAT) as safer supply and/or treatment [7, 9, 10]. With the onset of the pandemic in 2020, there has been rapid growth in the number of safer supply programs across Canada [7]. Early program evaluations have documented positive outcomes for participants, including decreased use of unregulated drugs and risk of drug toxicity deaths, improved physical health, improved social and economic well-being and reduced health care use and costs [6, 11–18].

Alongside research evaluating outcomes, there is a need for studies on implementation. Rapidly developed programs during a public health emergency provide a unique opportunity for research focused on understanding facilitators and barriers to implementation. There is a dearth of research examining service provider perspectives on implementation processes, reflecting on how safer supply programs emerged and shifted during COVID 19 and a co-occurring drug toxicity crisis. A previous study of professional stakeholders (i.e., program managers, health authority representatives) involved in the implementation of safer supply across Canada identified low-barrier models and participation in a community of prescribers as facilitators. Potential barriers included concerns about the temporary nature of programs, emphasis on treatment rather than harm reduction, lack of support from regulatory colleges, and the ongoing context of criminalization [19]. We contribute to this literature by exploring early implementation from the perspectives of an interdisciplinary team of service providers during the first year of implementation of one community-based prescribed safer supply program to provide in-depth insights related to program development.

The Safer Alternatives for Emergency response (SAFER) initiative is a flexible community based safer supply program, informed by harm reduction principles and staffed by an interdisciplinary team that includes outreach workers with lived/living experience, nurses, and physicians [20]. The target population includes people who use substances and are at high risk of overdose due to homelessness, low socioeconomic status, racism and colonialism, and a lack of connection with primary care [21]. Program participants receive pharmaceutical alternatives to the unregulated illicit drug supply alongside integrated supports for overall health and wellbeing, including those targeting systemic and structural drivers of inequities. The SAFER program (including both its prescribed medications and clinical encounters with prescribers) are covered by the government-run universal health insurance and accessed free-of-charge by participants.

The SAFER program was developed and implemented in May 2020, as a pilot program funded by Health Canada [20]. It is operated by AVI Health and Community Services, a non-profit health and social service organization supporting people who use substances, people living with HIV, and other marginalized populations in Victoria, BC [6, 11–18]. The SAFER program was implemented rapidly at the start of COVID 19, four years into a declared public health drug toxicity emergency in BC. Program funding coincided with the release of provincial interim clinical guidance, the Risk Mitigation Guidance (RMG), a form of safer supply prescribing of pharmaceutical alternatives to unregulated opioids, stimulants, and benzodiazepines [22]. When the initial cases of COVID-19 were detected in North America, the British Columbia Centre on Substance Use (BCCSU) rapidly developed the RMG to reduce the spread of COVID 19 and prevent overdose related deaths and harms among people who use drugs [22]. A recent population-based controlled study of the RMG, researchers found reductions in the rate of overdose mortality of 55–89% in the week following receipt of an opioid prescription meant to replace substances the unregulated drug market [23].

During the initial months of program development, the SAFER team drew on the RMG to inform early and rapid implementation. Early implementation focused on nurse-led outreach to people who use substances and living in city parks to connect them with a prescriber while consulting with services providers, and other stakeholders to inform program development [20]. The emerging SAFER team collaborated with a local drug user organization (SOLID Outreach) and academic researchers to conduct a study of the perspectives of people who use drugs on effective safer supply [24]. Further, the SAFER program utilized emerging data from their evaluation processes to rapidly implement program changes to better serve their clients.

Initially, as outlined in the RMG, prescribing focused on short acting hydromorphone tablets, but it quickly became apparent that this alone did not meet the needs of people who were regularly using fentanyl and its analogues. In fall 2020, the SAFER team (5 physicians, a clinical lead, project manager, and outreach workers) developed clinical workflows and prescribing protocols for oral oxycodone. Subsequently, the program expanded to include transdermal fentanyl patches and buccal fentanyl, as well as injectable sufentanil.

Through a community-based participatory research study, we explored experiences of implementation of the SAFER program from the perspectives of members of the SAFER interdisciplinary team engaged in program development and service provision during the first year of program operations. In so doing, we aim to generate insights into tensions and issues that service providers encountered and navigated during early implementation within a team-based approach to deliver safer supply.

## Methods

Nested within a community-based participatory research (CBPR) project, the study is evaluating SAFER program development, implementation, and impacts. The evaluation team includes representatives from a local drug user organization (SOLID Outreach), SAFER program leads, and academic researchers. Consistent with CBPR [25, 26], community partners were involved in all phases, from developing research questions and data collection tools, to interpreting data and knowledge dissemination. Additionally, the focus is on generating actionable evidence to support the development of flexible safer supply models.

Grounded in implementation science, this study centres on service provider perspectives. The Consolidated Framework for Implementation Research (CFIR) guided data collection and initial analysis. The CFIR provides a menu of 36 constructs arranged across five domains covering individual and intervention characteristics, implementation process, inner context and outer context [27, 28]. We used the CFIR to develop interview guides for service providers and as a sensitizing framework for data analysis (described below).

Semi-structured interviews were conducted with nine service providers on SAFER’s interdisciplinary team. The SAFER program employs 35 nurses, outreach workers, system navigators, and on-call physicians; such that approximately 25% (9/35) of staff were represented in our sample. We used purposive sampling to ensure representation of disciplines and roles. Respondents ranged from 27 to 50 years old. Gender identities are not disclosed to protect anonymity given the small sample size. The Research Ethics Boards of the University of Victoria and regional health authorities approved the study (Certificate number H20-01125) and respondents provided informed consent.

Interviews were conducted from April to September 2021 after one year of operation. They were audio-recorded and transcribed for analysis in NVivo. Data was analyzed using thematic analysis. Thematic analysis is a theoretically flexible approach to addressing complex experiential questions while producing practical outcomes [29, 30]. We followed Braun and Clarke’s six-phase process: familiarizing oneself with the data, generating initial codes, searching for themes, reviewing themes, defining and naming themes, and producing the report [30]. Two team members read transcripts (JM, BP) to review and identify key codes. The purpose of thematic analysis is to determine relationships between codes and inductively build a coherent whole through an iterative reasoning process. The goal is for patterns and relationships to become observable [31]. The research team met frequently throughout the study to review findings, solidify the results of the coding process, and support accurate interpretation.

### Findings

We identified six themes related to early SAFER program implementation and service delivery. As noted, early implementation of SAFER program coincided with the release of a related, but distinct province-wide clinical guidance document for prescribed safer supply (RMG). Themes reflect this complex context. The following sections describe the identified themes in detail: (1) RMG as a context for prescribing; (2) developing SAFER specific clinical protocols; (3) accessibility challenges and SAFER innovations; (4) interdisciplinary team and wrap around care; (5) program tensions between addiction medicine and harm reduction; (6) successes of safe supply and future visions.

#### RMG as a context for early SAFER implementation

As noted, the RMG (released in March 2020) enabled rapid development and implementation of the SAFER program. The SAFER team drew on RMG to guide initial delivery of the program as it was being developed for the local context. Both prescribers and other service providers reflected on the challenges, controversy and in some cases hesitancy to prescribe opioids as a replacement for the unregulated market, associated with the 2020 wider release of RMG across the province. One respondent recalled the sense of panic “*amongst physicians…’Oh my gosh, they released these guidelines and now we have to do this’ kind of dialogue… like a lot of apprehension and … I remember feeling very like “Oh my gosh, like this is such a step in the right direction.”* (2653) Another respondent said, “*I’m just waiting for my license to be threatened*.” (2652). These comments point to the concerns of prescribers about implementing risk mitigation prescribing in a politicized context where it was both necessary to address mortality from drug toxicity, and held the potential for professional risk.

Provincial controversy about risk mitigation prescribing was a challenge for SAFER program development. One respondent shared a perception that while many prescribers were reluctant, others embraced prescribed safer supply.

“…the guidance [RMG] had been intended so that any general practitioner, any, physician with access to a duplicate prescription pad could be able to pick it up and write a prescription to provide access to clean pharmaceutical grade alternatives to the toxic drug supply. Which, again, is a hopeful and ambitious effort, but there was no equity of access […] the vast majority of physicians don’t want to have anything to do with this. There’s a small group who do, but the problem is the small groups who do tend to be stuck in certain care provision contexts; that they are, clinically-based, they are tied to specific patient cohorts.” (2652)

This same respondent  discussed the rapid planning process that took place to develop the SAFER program, commenting, “*I have been lending my prescription pad*” (2652) to assist in mobilizing the program. This perspective reflects how a prescription pad is an important resource in the wake of high rates of unregulated overdose deaths, especially for the SAFER program’s target population.

In the context of new clinical guidance (the RMG), there was also fear that widespread prescribing might lead to unintended harms. One prescriber expressed the need to be aware of potential negative consequences of safer supply:

“… what are the harms? And I think that’s the part that’s really missing currently out of the data that’s being collected is tracking what are the harms and trying to figure out, okay, what are the rates of new onset opioid use disorder from [hydromorphone] pills, for example. In addition to like the overdose deaths related to [hydromorphone] pills […] And those are the bits, that from a prescriber’s perspective, are the reason it’s so anxiety provoking, is because we don’t know.” (2655)

Prescriber concerns about the unintended consequences of RMG affected initial SAFER program development manifesting as concerns and discomfort with prescribing. These concerns highlight a legacy of overprescribing of opioids as a driver of overdose deaths with early waves of overdoses in the United States and elsewhere attributed to prescription drugs [32, 33]. In Canada, there is ongoing monitoring and surveillance of overdose mortality and prescribing. Pre-pandemic, reports highlighted that prescribed opioids are rarely detected alone as a cause of toxic drug deaths [34, 35]. Toxicology analyses continue to implicate fentanyl from the unregulated drug supply as the main driver of overdose deaths, with hydromorphone implicated in less than 2% of overdose deaths in BC from March 27, 2020 to May 31, 2021 [3, 36]. The concerns of prescribers with the SAFER team, nonetheless, speak to the tensions involved in developing and implementing medicalized models of safer supply in a context of broader efforts to reduce opioid prescribing (e.g., clinical guidelines for chronic pain management and intensifying prescription monitoring programs) [37–39]. Addressing such tensions and concerns during early SAFER program implementation was important to ensure prescribers were available and willing to prescribe.

#### Developing SAFER specific clinical protocols

With concerns about prescribing pharmaceutical alternatives to unregulated drugs, as well as the fit of the medications in the RMG for the target population, a key SAFER program activity involved the iterative development of SAFER-specific clinical protocols outlining medications and dosages. In reflecting on the importance of program specific clinical protocols, respondents spoke to the perceived inadequacy of RMG prescribing for replacing the unregulated market. Referring to the medications in the Guidance, one respondent stated, *“…they didn’t hit the mark.”* (2641). Another reflected:

“The advice given [in the RMG] around 14 tablets of [hydromorphone]…seems to be quite an arbitrary number. And it seems to be quite a hopeful number, but it doesn’t seem to be a very effective number.” (2652)

Respondents noted the discrepancy in potency between tablet hydromorphone and unregulated fentanyl, and connected this to an inability to meet SAFER program participants’ needs. As one respondent put it: *“we’re not giving them their drug of choice and we’re not giving them the high that they want*.” (2653). According to respondents, prescribing under the RMG was not providing the right drugs at the right dose for the target population: “*what is available through the risk mitigation guidance documents are being vastly outpaced by people’s growing tolerances. And 14 tabs of [hydromorphone] for many is a tic tac, and it’s not effective.”*(2641). In addition to the limited types of medications and inadequacy of the dosages for some participants, respondents cited challenges associated with the routes of administration of the available medications.

“There is no consideration for a smokeable version. When you look at what substances were provided and what substances are suggested and the dose ranges, it doesn’t speak to the fact that many people smoke their drugs, and many people who – the majority of British Columbians who are dying by overdose are people who smoke their drugs. And those aren’t options that are available.” (2641)

In response, the SAFER team developed prescribing protocols, expanding on the RMG to incorporate oxycodone initially and then prescription fentanyl and other options. One respondent stated, *“the things that we’re trying to protocolize right now are fentanyl-based safe supply interventions.”* (2652).This allowed for greater flexibility and options for SAFER program participants. As one respondent explains:

“I think some doctors may take it as like set in stone instead of just as a guideline for them to use if they wish […] As for effectiveness, I mean, no doctors really other than our doctors are prescribing safe supply without OAT, so it’s really…I don’t know. I don’t know how effective it is in that way.” (2688)

While viewed as a necessary step, the development of clinical protocols for the SAFER target population also presented challenges, including perceived censure by colleagues. One respondent shared:

“So as soon as I started prescribing... I tried those things [options in the RMG] and found out that, no, they didn’t work that good, So I tried some other things. And I immediately was accosted by my colleagues who were saying ‘Well that’s not in the guidance documents. You can’t use those [medications]. And I said ‘Why not?’ […] the guidance document is only guidance […] I’m just trying whatever we can use to see if we can make a difference.” (2652)

Importantly, the SAFER program protocols were developed in part in response to community needs and in partnership with people who use drugs: “*People with lived experience were a key part of this process.*” (2654). During the program development phase, key elements of effective safer supply identified by service users included the importance of the right drug and right dose as part of effective safer supply [24].

#### Accessibility challenges and SAFER innovations

Respondents noted that the SAFER program’s target population faced multiple barriers to accessing a prescribed safer supply program, including stigma, program capacity limits, homelessness and continuing displacement from encampments, tensions between OAT and safer supply, and program costs. We describe these barriers below, along with strategies used by the SAFER team during program development and implementation. These strategies, along with development of the program-specific clinical protocols, highlight the innovation and adaptability of the program to align with the needs of their local target population.

Respondents recognized that SAFER program’s target population often faces high levels of drug-related stigma when accessing services and that understanding and overcoming that stigma is essential for building safety and trust.

“So there’s a lot of people who just wouldn’t access because maybe, you know, they’ve been – they’ve had so many issues with the healthcare system that they don’t necessarily believe that we have this great group of outreach people who are going to advocate for them, right?” (2655)

The SAFER program used outreach as a key innovation to address these accessibility barriers. Respondents described the effort put in by the team to reach participants early on during the pandemic:

“Outreach to encampments, to homeless encampments, and seeing if we could engage some of the most marginalized and disenfranchised people in the country to be able to access a care intervention that is supposed to be accessible to all.” (2652)

In line with harm reduction principles, the team brought the care to participants instead of expecting them to show up at a clinic or pharmacy to enhance program accessibility. “*So making it to the pharmacy every day is impossible. Making it to a doctor’s office is impossible. The care had to be brought to the people and the medication delivery had to be brought into the homeless encampments…*” (2652). However, outreach workers faced challenges due to external issues such as displacement and disruptions to encampments.

“…so we have to go and find people. And often times the city and police and bylaw are evicting people from homeless encampments and displacing them from homeless encampments, and every time that happens, we lose people to contact, people get lost in the shuffle, and we have to spend a lot of time and energy trying to find them again. Ah, because if their prescription lapses or if they’re not able to access their meds, then there’s a very heightened risk period for them.” (2641)

This respondent observes the heightened risk of overdose when service users are not able to access their medications, which aligns with evidence linking opioid safer supply to reduced overdose mortality [23].

Respondents shared that the SAFER program quickly reached capacity and was unable to take new participants. Put simply by one respondent: “*there is literally no more space in the program,”* (2652). While incorporating outreach to participants reduced barriers to access, barriers related to the external environment, including displacement of encampments and limits of program size remained a challenge.

Respondents further commented on accessibility challenges caused by lack of integration between safer supply and OAT prescribing outside of the SAFER program. One respondent provided the following example of the challenges faced when an OAT prescriber did not support prescribed safer supply:

“You cannot be already linked with another OAT prescriber, which has been a huge barrier for a lot of folks who have prescribers […] like there are some prescribers that are just straight up not prescribing, any safer supply.” (2653)

This respondent is highlighting that some OAT prescribers would not prescribe safer supply for those on OAT and may discontinue OAT for those receiving safer supply. Prescribers on the SAFER team overcame this barrier by offering safer supply mediations with and without OAT as individually and clinically appropriate for each patient.

Despite being covered by provincial universal health insurance, the loss of government-issued identification and housing instability can lead to lapses in insurance coverage for individuals. Respondents noted that this can lead to SAFER program participants incurring upfront costs for their safer supply medications.

“We have people who don’t have [government insurance], who don’t have… ID; and so you can write the script, but then they can’t pay for their medications. So our project covers the cost of the medications while our systems navigators does the ID and birth certificate application and gets the [government insurance]. And then once that’s done, then we stop paying for it and it gets covered, through their [government insurance].” (2641)

In these cases, respondents noted that the SAFER program would incur the costs of the medications to remove this barrier to access.

Through targeted innovations and flexibility (including outreach, prescribing with/without OAT and covering costs incurred by program participants), the SAFER team sought to address barriers that affected their target population. Other issues, such as displacement of encampments where participants were living and program capacity limitations were beyond their control and remained salient challenges through the first year of the program.

#### Interdisciplinary team and wrap around care

Respondents spoke about how they worked to provide safer supply and to implement supports for other aspects of participants’ lives to ensure ongoing access and participant stability.

“…and then, just try to get them in a more stable situation from a medical medication side of thing. And, but then still connect them to housing, and tax services, and all these other things. Like just see if they need something, and we’ll connect them to something. Um, and, and we’ll do wound care and stuff as needed. Um, because we’re there with the nurse.” (2656)

This work included connecting participants with primary care and social services: “*We try to hook people up with primary care through us or through elsewhere, and we try to have them access housing or income support….”* (2668). Respondents described this as putting the pieces together.

SAFER team members spoke about their experiences helping participants to navigate complex tasks, such as obtaining or replacing identification and applying for income and housing supports.

“I’ve had to fill out applications for all sorts of things, and it’s like, my god, if I didn’t have a, you know, filing cabinet with some of this stuff, like how do you find half of these things? Or if you don’t have – if you’re no fixed address and you’re filing for a birth certificate and it says “Where are we sending this to?” Where do you send it to? You know what I mean? Or just having access to a computer to do all of these things. Or even the payment for the birth certificate, right? Like it’s just all of these little things are barriers to just getting ID.” (2667)

Wraparound health and social supports were critical elements of the SAFER program essential to meet the needs of the target population.

#### Program tensions between addiction medicine and harm reduction

As part of implementation, respondents commented on what they saw as an emerging tension within the program between harm reduction and addiction medicine principles and practices. In the broader context of the RMG provincially, prescribers were directed to and drew on established addiction medicine practices such as daily dispensing and witnessed dosing OAT medications, and urine drug screens. Within the SAFER program, which was being implemented within an organization with a long history of harm reduction, these practices naturally created tensions, as interdisciplinary team members brought forth differing perspectives. One respondent noted in a critical reflection of the addiction medicine approach:

“Embedded within addiction medicine is a number of very paternalistic practices. And it is about control and surveillance. And that means urine drug screens, that means daily pick-ups, that means treatment adherence follow-ups, conditionalizing OAT as a requirement to get on to safe supply. Those are the things that don’t work.” (2641)

Similarly, another respondent noted that:

“Like I don’t know if this is exactly part of that document [RMG], but I know people still […] have to do urine drug screens and that kind of stuff. And […] it’s a thing of not having agency and feeling like you’re untrustworthy.” (2656)

This respondent problematizes these practices as reducing the agency of program participants and working against the development of trust.

Others described these tensions in terms of differences between top-down and bottom-up approaches to program development and implementation:

“So if you put like a blanket guidance, then you’re always going to have issues, right? And I think that that kind of paternalistic controlling nature is part of a new project unfortunately. But yeah, it would be nice to have the people on the ground level who are interacting with the clients make those decisions as opposed to kind of more of a blanket from the higher ups.” (2667)

Here, the respondent shares a perspective that brings forward an inherent tension between harm reduction (as a bottom-up approach that emerges from service users' perspectives) and addiction medicine providers (as a more top-down approach).

These tensions affected early implementation of the SAFER program in various ways. Several respondents connected prescribing with power given the authority associated with writing prescriptions: “*the doctors have the final say, or more say than everybody else, because of the power of writing a prescription.*” (2653) This highlighted the perspective of team members that, because of their unique role and power, prescribers ultimately got to decide how the SAFER program should operate, including what medications are prescribed and how. Others described how centering program participants in decision-making was critical to a harm reduction approach, upholding individual agency and autonomy:

“I am client-led. If they want to do something, then great. If they don’t, then, you know, that’s their decision. Harm reduction is about putting the client first. Ah, and just ensuring that they are as safe as possible in whatever way that you can. But at the end of the day, it’s their right to live at risk if they so choose. And how can we help support that person to live at a smaller risk as possible? […] It’s about keeping people as safe as they can be while accepting – while respecting their autonomy as an individual human to make their own decisions and choices and live at risk in the way that they so choose. But to be there to support them when they decide that they want to make a shift in one way or the other, and to support them in whatever way they need or choose that they tell you that they need.” (2668)

Another respondent emphasized safer supply as evidence-based practice and client-centered care:

“Safer supply is part of client-centred care. It is using knowledge from research to support someone to access appropriate medication and to, you know, prevent harms, that are associated with the illicit market.” (2667)

The next respondent highlights how the harm reduction orientation of the lead organization was an important factor influencing implementation:

“I think that they [AVI Health and Community Services] were very prepared. They’ve been doing radical kind of frontline work, harm reduction work, for a very long time, and I think that the people that work there all have wanted this for a really long time. And I think that they’re very capable, and I think that they have the right spirit to get it done and to fight for it and to move it forward.” (2667)

The tension between a harm reduction philosophy that centres the person and focuses on providing alternatives to the unregulated drug supply, and medical models of safer supply that position the prescriber as the authority emerged during early SAFER program implementation. Balancing this tension meant reconciling addiction medical principles and practices with harm reduction principles and practices that uphold individual autonomy.

#### Successes of safe supply and future visions

Respondents highlighted their perspectives on the qualitative impacts of the program and key indicators of program success. Multiple respondents described how program participation protected against overdose, despite rising community overdose rates during the period of program implementation: *“none of our clients have overdosed and died on our, on safe supply. And like that’s a win right there. That’s a big win.”* (2668).

Many respondents shared that program participants had either stopped taking illicit drugs or had reduced their use. As one respondent reported:

“None of our clients have died. And during the worst iteration of BC’s overdose crisis and the fact that we’re working with the most marginalized and made vulnerable by the system people, that’s low hanging fruit, but it’s the most important low hanging fruit that you can have. We also have people now who provide urine drug screens that have zero fentanyl in it, and they’ve stopped using any of the illicit drug supply. We also have people who report to us that they’re using 50% less of what they previously used.” (2641)

Another respondent stated, “*I would have to say eight out of ten folks are, really receptive to the program. Um, their, their use has gone down dramatically.”* (2671). Another noted success was around the engagement of people who had previously been reluctant to seek health care. *“This new option has been really useful in engaging them in care.”* (2654) Engagement in the SAFER program was seen to, in turn, facilitate access to a broad array of healthcare: *“It’s been helpful in providing a lot of Hep C treatment to individuals, like, yeah just making sure that – cause I think a good part of it is rapport with the clinic itself.”* (2654).

Respondents further described how program participation positively impacted people’s lives:

“I have a handful of patients who are […] picking up their meds regularly – accessing the team regularly, reporting that they’re using quite a bit less than they were, finding that they’re able to reconnect with their families. Um, some of them have been able to get back to work here and there; maybe not full-time, but part-time. That kind of stuff.” (2655)

One of the respondents reported that a *“lot of the folks are finding housing.”* (2671) Another related how not being so reliant on illicit drugs had helped participants with their daily routines: *“A lot of [participants] are saying that it just gives them a little bit more breathing space in their day to not have to be, constantly shopping.” (*2688*)*.

While respondents were instrumental in early implementation during exceptionally trying circumstances (corresponding to the beginning of the COVID-19 pandemic and related service closures and disruptions), they also envisioned and were working iteratively toward program improvement. Several respondents felt that the program should move toward offering a continuum of models of safer supply in addition to a medicalized, prescriber-driven model. As one respondent said:

“The real crux of safe supply is that there’s no one model that’s going to be effective. People need choices and they need options depending on where they’re at. Some people will benefit from seeing their doctor. Some people need heroin compassion clubs. Some people need to just go to the pharmacy and fill out some paperwork. Some people need iOAT, TiOAT. It needs to be a whole continuum.” (2641)

Some respondents felt that reducing barriers to safer supply might require not involving prescribers at all:

“I would like it [substance] just to be like a lot more low barrier. Just kind of like, I don’t know, almost like in how you get, you know, like something informed consent and you can just buy it, and you know the risks.” (2656)

These visions for the future of the SAFER program connect with the above themes around the tensions between harm reduction and addiction medicine and articulate several potential future directions for the program, as the interdisciplinary team seeks to address ongoing barriers to access.

## Discussion

In the development of the SAFER program, service providers highlighted issues related to the RMG as a context for early program implementation, the need for programmatic clinical protocols to meet participant needs, addressing barriers to accessibility, tensions between addiction medicine and harm reduction, and successes. Key components to make the SAFER program more accessible were outreach and interdisciplinary teamwork. Additional program elements related to the social determinants of health were crucial to supporting the SAFER target population, as adjuncts to providing prescribed safer supply. A separate study on implementing prescribed safer supply indicated that client instability is a factor preventing adherence to medications [40] and called for additional supports to enhance adherence.

Though prescribers in the program were committed to providing safer supply, they were also practicing in a professional context that was not always supportive of prescribed safer supply. Reluctance to prescribe is not unique to safer supply. In a 2014 study, Hutchinson et al. found that prescribers were reluctant to prescribe buprenorphine because of a perceived lack of institutional support and little in the way of mental and psychosocial support [41]. Nonetheless, prescribers with the SAFER program were operating within a particularly politicized context heightened during the dual public health emergencies in BC throughout 2020-21. This context came through in the experiences and reflections of prescribers involved in the implementation and operation of the SAFER program.

Accessibility challenges are a recurrent barrier to safer supply implementation. Access to a primary care physician or nurse practitioner is essential to acquiring a safer supply prescription. In BC, only 82% of the population have access to a primary care provider, which is below the national average [42]. Prescribers who are less knowledgeable about opioid use disorder are far less likely to be willing to prescribe OAT, and compared to addiction specialists, general practitioners tend to hold more negative attitudes towards people who use drugs [43, 44]. Within the context of the SAFER program, our findings highlight how an appreciation of these barriers was translated into practices designed to meet the needs of the target population. For example, respondents attempted to reduce barriers by [45]prescribing safer supply both with or without OAT based on participants’ needs and preferences.

The SAFER team took into consideration BC’s provincial clinical guidance for risk mitigation prescribing (a form of safer supply) when developing program-specific clinical protocols. Our findings highlight the ways that the SAFER team interpreted and adapted the guidance to their specific patient cohort, expanding medication options and dosages [14]. Evaluations of safer supply programs across Canada have highlighted the need for a broad array of medication options, in terms of type of drug (e.g., opioids, stimulants) and mode of use (e.g., inhalation, injection, oral) [5, 16–18, 45]. By iteratively developing clinical protocols, the SAFER program was able to offer a range of medication options at doses that meet the local context of the unregulated drug market and, consequently, the needs of the target population [21].

Throughout program development and implementation, the SAFER team was navigating the integration of a community-based harm reduction approach with a medicalized prescription-based model. Given prohibition, the medical model is currently the dominant approach to providing safer supply in Canada. Yet, people who use drugs are often reluctant to seek health care because of histories of experiencing stigma from service providers. Petrasko (2022) posits that the medical model has become the default process for delivering safer supply because the legitimacy of prescribers’ work makes safer supply more palatable to politicians, the public and police forces [46]. However, many health care professionals are either reluctant to prescribe safer supply or they prescribe less potent drugs that do not adequately meet participants’ needs [46]. In the United States, medicalized cannabis programs have been observed to have lower enrollment compared to non-medicalized programs [47].

These issues, manifested in respondent perspectives on the tensions between addiction medicine and harm reduction perspectives within the delivery of safer supply programs, have been reported in other contexts as well [48]. To be sure, harm reduction-oriented addiction medicine is practiced and has been associated with positive outcomes, such as increased referrals to needed supports and services, reduced stigma, and increased knowledge of safer use practices [49, 50]. At the same time, critics have noted how harm reduction principles are overshadowed when co-opted by mainstream health services because of medical hegemony [51]. Results include the privileging of provider expertise rather than centring the authority of people who use drugs, contrary to the initial radical grassroots nature of harm reduction. Within the SAFER program, a key implementation challenge was balancing addiction medicine principles and practices with harm reduction principles and practices given the history of the lead organization. The use of prescriptions made it possible for participants to access safer supply and the harm reduction orientation of the organization, while being in tension with addiction medicine principles, also meant that the resultant program was community driven. As a grassroots, community-centered organization, AVI Health and Community Services demonstrated ability to be flexible and responsive to client needs, and by doing so, pushed back against top down, medicalized and institutional approaches in developing the SAFER clinical protocols and program innovations.

In a review of Canada’s safer supply programs [52], reported issues related to program capacity, medicalization, challenges related to addressing health inequities and limited organizational resources. Innovations in the SAFER program were designed to remove barriers to access through an interdisciplinary team supporting participants to access safer supply alongside other health and social supports. Reliable teamwork and collaboration is known to be critical to delivering quality care [53]. The SAFER program model emphasized outreach and made efforts to deliver medications in the community, instead of requiring participants to come to the program. This nimble approach, which emphasized inclusionary and innovative practices, was an alternative to what are often lengthy and onerous sanctioning practices [54]. However, some challenges such as the continuing displacement of homeless encampments and program size were beyond their ability to change.

These findings contribute to understanding program development within a broader provincial initiative to introduce risk mitigation prescribing (RMG). Learnings from the SAFER program informed the BC prescribed safer supply policy direction released in 2021 [55]. Specifically, this provincial policy direction expanded the list of available medications and dosages that were previously included in the RMG. At the program level, SAFER expanded dose ranges and opioid options to provide oxycodone and regulated fentanyl, both in the form of patches and in oral form, as well as Sufentanil, which can either be injected or taken sublingually [20, 56]. Ongoing evaluation and learnings from SAFER continue to be shared widely with funders and policymakers as a means to advocate for regulatory changes that expand availability and accessibility of safer supply, while also promoting non-medicalized, community-led programs.

This study captures perspectives from service providers at one safer supply program. Findings may not be transferable to other programs, locations or jurisdictions, and other points in time. This study focused on early implementation of one of the first safer supply programs in BC, developed during the dual public health emergencies of COVID-19 and the ongoing overdose-related public health emergency since 2016. Service provider findings are reported in this study as part a larger mixed methods study that also captured service user perspectives on implementation and impacts. Given the essential roles of service providers in making safer supply accessible to people who use drugs, this study adds to knowledge for developing safer supply programs for people at high risk of overdose by a harm reduction organization. Further research with service providers is needed to better understand and facilitate implementation of safer supply in diverse contexts.

## Conclusion

Service providers with the SAFER program supported safer supply as a life-saving harm reduction intervention to prevent drug toxicity deaths. Barriers to program implementation included concerns and anxiety of prescribers given the politicized nature of safer supply introduced during the dual public health emergencies of COVID-19 and overdose-related deaths. Program-specific clinical protocols allowed the SAFER program to adapt to the local context, offering a range of medications and dosages to align with participants’ needs. Additional program innovations were developed to overcome the specific challenges experienced by the target population (e.g., need for outreach, coverage of medication costs, and wraparound care). However, some barriers such as encampment displacement and limits to program capacity remained. Lessons learned from the process of implementing the SAFER program reflect larger tensions between addiction medicine and harm reduction around prioritizing client centredness and personal agency when providing medicalized prescribed safer supply. The development of SAFER clinical protocols and program innovations centred client needs prioritizing individual and community input into program development and implementation.

## Data Availability

Data is drawn from qualitative interviews and due to the sensitive nature, raw data is not available.

## Abbreviations

RMG: Risk Mitigation Guidance
SAFER: Safer Alternatives for Emergency Response
AVI: AVI Health and Community Services
CBPR: Community Based Participatory Research
CFIR: Consolidated Framework for Implementation Research
OAT: Opioid Agonist Therapy
